# Sleep quantity and quality during heat-based training and the effects of cold-water immersion recovery

**DOI:** 10.1186/2046-7648-4-S1-A150

**Published:** 2015-09-14

**Authors:** Geoffrey M Minet, Rachel Gale, Georgia Wingfield, Frank E Marino, Tracy L Washington, Melissa Skein

**Affiliations:** 1 School of Exercise and Nutrition Sciences, Queensland University of Technology, Brisbane, Australia; 2 Institute of Health and Biomedical Innovation, Queensland University of Technology, Brisbane, Australia; 3 School of Human Movement Studies, Charles Sturt University, Bathurst, Australia; 4 Civil Engineering and Built Environment School, Queensland University of Technology, Brisbane, Australia

## Introduction

Heat-based training (HT) is becoming increasingly popular as a means of inducing acclimation before athletic competition in hot conditions and/or to augment the training impulse beyond that achieved in thermo-neutral conditions. Importantly, current understanding of the effects of HT on regenerative processes such as sleep and the interactions with common recovery interventions remain unknown. This study aimed to examine sleep characteristics during five consecutive days of training in the heat with the inclusion of cold-water immersion (CWI) compared to baseline sleep patterns.

## Methods

Thirty recreationally-trained males completed HT in 32 ± 1 °C and 60% rh for five consecutive days. Conditions included: 1) 90 min cycling at 40 % power at VO_2max _(P_max_) (90CONT; n = 10); 90 min cycling at 40 % P_max _with a 20 min CWI (14 ± 1 °C; 90CWI; n = 10); and 30 min cycling alternating between 40 and 70 % P_max _every 3 min, with no recovery intervention (30HIT; n = 10). Sleep quality and quantity was assessed during HT and four nights of 'baseline' sleep (BASE). Actigraphy provided measures of time in and out of bed, sleep latency, efficiency, total time in bed and total time asleep, wake after sleep onset, number of awakenings, and wakening duration. Subjective ratings of sleep were also recorded using a 1-5 Likert scale. Repeated measures analysis of variance (ANOVA) was completed to determine effect of time and condition on sleep quality and quantity. Cohen's d effect sizes were also applied to determine magnitude and trends in the data.

## Results

Sleep latency, efficiency, total time in bed and number of awakenings were not significantly different between BASE and HT (P > 0.05). However, total time asleep was significantly reduced (P = 0.01; d = 1.46) and the duration periods of wakefulness after sleep onset was significantly greater during HT compared with BASE (P = 0.001; d = 1.14). Comparison between training groups showed latency was significantly higher for the 30HIT group compared to 90CONT (P = 0.02; d = 1.33). Nevertheless, there were no differences between training groups for sleep efficiency, total time in bed or asleep, wake after sleep onset, number of awakenings or awake duration (P > 0.05). Further, cold-water immersion recovery had no significant effect on sleep characteristics (P > 0.05).

## Discussion

Sleep plays an important role in athletic recovery and has previously been demonstrated to be influenced by both exercise training and thermal strain. Present data highlight the effect of HT on reduced sleep quality, specifically reducing total time asleep due to longer duration awake during awakenings after sleep onset. Importantly, although cold water recovery accelerates the removal of thermal load, this intervention did not blunt the negative effects of HT on sleep characteristics.

## Conclusion

Training in hot conditions may reduce both sleep quantity and quality and should be taken into consideration when administering this training intervention in the field.

